# MRI characteristics are predictive for CDMS in monofocal, but not in multifocal patients with a clinically isolated syndrome

**DOI:** 10.1186/1471-2377-9-19

**Published:** 2009-05-20

**Authors:** Jessica M Nielsen, Christoph Pohl, Chris H Polman, Frederik Barkhof, Mark S Freedman, Gilles Edan, David H Miller, Lars Bauer, Rupert Sandbrink, Ludwig Kappos, Bernard MJ Uitdehaag

**Affiliations:** 1 MS Center, Department of Neurology, VU Medical Center, Amsterdam, the Netherlands; 2 Bayer Schering AG, Berlin, Germany; 3 Department of Radiology, VU medical Center, Amsterdam, the Netherlands; 4 Department of Neurology, The Ottawa Hospital, Ontario, Canada; 5 Department of Neurology, Hopital Pontchaillou, Rennes, France; 6 Department of Radiology, Queen square hospital, London, UK; 7 Department of Neurology, Kantonsspital, Basel, Switzerland; 8 Department of Epidemiology and Biostatistics, VU Medical Center, Amsterdam, the Netherlands

## Abstract

**Background:**

To diagnose multiple sclerosis (MS), evidence for dissemination in space and time is required. There is no clear definition on how symptoms and signs of a patient indicate clinical dissemination in space. To provide a uniform approach on this subject, a clinical classification system was described recently differentiating patients with mono- and multifocal clinical presentation. Here we assess the predictive value of clinically defined dissemination in space at first presentation for time to clinically definite MS (CDMS).

**Methods:**

Four hundred and sixty-eight patients with a first episode suggestive of MS were classified as clinically mono- or multifocal by two neurologists blinded to magnetic resonance imaging (MRI) results. These patients were part of the BENEFIT study in which 292 patients were randomized to interferon beta-1b (IFNB-1b) and 176 to placebo. By using Kaplan-Meier statistics the risk for CDMS was studied in mono- and multifocal patients of the placebo group, both with and without taking into account MRI measures of potential prognostic relevance.

**Results:**

Time to CDMS was similar in monofocal and multifocal patients. In monofocal patients, the risk for CDMS over 2 years was significantly higher when ≥ 9 T2 lesions or at least one Gd-enhancing lesion were present at the first event or 3 or 6 months after the first event. In patients with multifocal presentation, these MRI measures had no significant added value in predicting time to CDMS.

**Conclusion:**

These data indicate that a carefully performed neurological assessment of symptoms and signs, combined with lesions on MRI, is important for defining the risk of conversion to CDMS.

**Trial Registration:**

The Benefit trial has been registered under NCT00185211

## Background

Multiple sclerosis (MS) has a highly variable disease course[1] and knowledge of factors that predict subsequent disease course in individual patients with a first event suggestive of MS (also called patients with a clinically isolated syndrome: CIS) is limited.

In CIS patients, magnetic resonance imaging (MRI) characteristics have been described as a predictor of conversion to clinically definite MS (CDMS) and of the subsequent disease course. CIS patients with an abnormal cerebral MRI scan at presentation have a substantially higher long-term risk of conversion to CDMS than those with a normal cerebral MRI[2]. Diagnostic guidelines for MS include detailed MRI rules for the definition of dissemination in space of MS-specific pathology.[3,4] In untreated[5] and treated[6] CIS patients fulfillment of these criteria is associated with a high risk of CDMS.

According to the recommendations of the International Panel (IP) on the Diagnosis of Multiple Sclerosis, disease dissemination in CIS patients can also be identified by clinical examination of symptoms and signs at the first clinical event.[3] In contrast to a detailed algorithm on the use of MRI criteria, however, it was left unclear how clinical disease dissemination should be evaluated. Recently it was shown that the clinical assessment of disease dissemination can vary widely between physicians.[7] To standardize these assessments, a clinical classification system was proposed.[7] This system was centrally applied to patients of the BEtaferon^®^/BEtaseron^® ^in Newly Emerging multiple sclerosis For Initial Treatment (BENEFIT) study, a study evaluating the impact of interferon beta-1b (IFNB-1b) in CIS patients. By analyzing baseline data from this study we have recently shown that patients with clinical dissemination in space (multifocal, indicating more than one clinical lesion) had more lesions on their MRI than monofocal patients (those exhibiting symptoms and signs from only one clinical lesion).[8]

In the present study we assessed the prognostic value of this clinical classification system[7] for conversion to CDMS and the added value of potentially prognostic MRI parameters, by analyzing data obtained during the placebo-controlled treatment period of the BENEFIT study.

## Methods

### Study design, patients, and procedures

BENEFIT is a multicenter study comparing IFNB-1b to placebo in CIS patients for up to 2 years, followed by a follow-up period with IFNB-1b for up to 5 years after the CIS. For the present analyses we used data from the placebo-controlled first 2 years of the study. The design and main outcomes of the placebo-controlled phase of the BENEFIT trial have been reported elsewhere[9]. Patients completed the placebo-controlled phase of BENEFIT if they either reached 24 months of follow-up or were diagnosed with CDMS. Briefly, inclusion criteria encompass: age between 18 and 45 years, presentation with a first neurological event suggestive of MS, and the presence of at least two clinically silent lesions on a T2-weighted brain MRI scan with a minimum size of 3 mm, at least one of which was ovoid, periventricular, or infratentorial.

Patients were randomly assigned in a 5:3 ratio to IFNB-1b 250 μg or placebo, by subcutaneous injection every other day. Study treatment was initiated within 60 days of confirmation of the first clinical event. Regular visits were scheduled for collection of clinical, MRI findings, and other data on disability progression as measured by the expanded disability status scale (EDSS)[10], All MRI scans were performed with 0.1 mmol/kg gadolinium. MRI findings and other parameters at months 3, 6, 9, 12, 18, and 24. Several MRI parameters were analyzed; in particular, number of: gadolinium (Gd)-enhancing lesions, hyperintense T2 lesions, hypointense T1 lesions, and newly active lesions (NALs). A NAL was defined as a new T2 hyperintense or Gd-enhancing lesion, or a newly enlarging T2 lesion. The numbers and volumes of hyperintense lesions on T2-weighted images and Gd-enhancing lesions on T1-weighted images were centrally evaluated at the Image Analysis Center in Amsterdam, The Netherlands. MRI analyses were performed by expert readers who were blinded to the patients' clinical classification.

As MS according to the criteria proposed by the IP on the Diagnosis of Multiple Sclerosis[3] was one of the primary outcome measures, much emphasis was placed on the clinical classification of patients. On the basis of all available information of clinical signs and symptoms, as documented by the local investigator, patients were classified centrally by the consensus of two neurologists (CHP and BMJU) as having an either monofocal or multifocal disease presentation according to the previously described standardized scheme.[7] Briefly, on the basis of the neurological symptoms the minimum number of central nervous system (CNS) areas that could explain all symptoms was determined (= monofocal or multifocal presentation as defined by symptoms). Subsequently, it was decided whether abnormalities as revealed by the neurological examination (= signs) indicated the presence of additional lesions in the CNS (= monofocal or multifocal presentation as defined by signs).

In the case of a multifocal presentation it was then decided whether the patient's multifocal classification was purely based on multiple presenting symptoms (these patients are denoted as multifocal patients by symptoms) or whether clinical signs indicated additional CNS lesions that did not correspond to any of the presenting symptoms (these patients are denoted as multifocal patients by signs). This subclassification of multifocal patients was carried out under the hypothesis that such additional clinical signs (in the absence of concomitant symptoms) might point to subclinical disease activity preceding the reported onset.

### Statistical analysis

The following analyses were performed to evaluate different disease characteristics of 1) monofocal versus multifocal CIS patients; and 2) multifocal CIS patients by signs only versus by symptoms only. In order to avoid any influence of IFNB-1b treatment on the results, analyses on time to CDMS were only performed in placebo patients whilst analyses on baseline parameters were performed in the total patient cohort. Analyses were performed using SAS.

### Comparison of key baseline characteristics between mono- and multifocal patients at the first event and between the multifocal subgroups by signs and by symptoms (all patients)

The following parameters were analyzed: Age, sex, steroid use at first event, EDSS at screening, positive CSF findings, number of T2-hyperintense lesions, number of/proportion of subjects with at least one Gd-enhancing lesion(s), proportion of subjects with at least one T1 hypointense lesion.

### Comparison of time to CDMS and MRI disease activity between the monofocal and multifocal patients and between the multifocal subgroups by signs and by symptoms (placebo patients)

Time to CDMS was analyzed as a measure for clinical activity and the annualized cumulative number of NALs over the study was analyzed as a measure of subclinical activity.

### Comparison of the impact of MRI findings at screening, month 3, and month 6 on time to CDMS within the monofocal and multifocal group separately (placebo patients)

These analyses were only performed on data from the mono- and multifocal subgroups, as patient numbers were too small to further stratify the multifocal subgroups by symptoms and by signs. The following MRI parameters were evaluated: presence of Gd-enhancement, or pronounced disease dissemination (≥ 9 T2 lesions) on the screening MRI; new Gd-enhancement at months 3 or 6.

Dichotomous variables were compared using Fisher's exact test. Continuous variables were compared using the Mann-Whitney U-test (comparison of the cumulative number of NALs was done by a Wilcoxon test). Kaplan-Meier survival analysis was used to analyze time to CDMS. Group comparisons for this outcome measure were performed using the log-rank test. Interaction of clinical mono-/multifocality and MRI parameters was analyzed by Cox proportional hazards regression. Reported p-values are based on two-tailed significance tests, with the threshold for significance set at 0.05. Analyses were performed post hoc on data from the placebo-controlled period of the BENEFIT study for all patients who were randomized and received study medication at least once.

## Results

Four hundred and eighty-seven patients were randomized and 468 started treatment in the BENEFIT study. Four hundred and thirty-seven of these (93.6%) completed the placebo-controlled study. Two hundred and ninety-two patients received IFNB-1b and 176 received placebo. The main outcome data have previously been published[9].

### Comparison of key baseline characteristics of mono- and multifocal CIS patients (all patients)

Two hundred and forty-six (53%) patients were classified as monofocal and 222 (47%) as multifocal. Baseline characteristics of mono- and multifocal patients are outlined in Table 1 and have been reported previously[8]. In summary: Multifocal patients had a higher number of T2-hyperintense lesions (p = 0.018) and more frequent T1-hypointense lesions (p = 0.030).

**Table 1:** Disease characteristics of monofocal vs multifocal CIS patients

	All	Monofocal	Multifocal	p-value*
N	468 (100%)	246 (53%)	222 (47%)	

Sex – % of females	71%	66%	76%	**0.0325**^†^

Age, median (quartiles)	30 (24–37)	29 (24–37)	31 (25–37)	**0.0820**^‡^

Steroid treatment – %	71%	72%	70%	0.6835^†^

EDSS (screening), median (quartiles)	2 (1–2.5)	1.5 (1–2)	2 (1.5–2.5)	**< 0.0001**^‡^

CSF positive of samples taken – %	267/314 85% (57%)	149/176 85% (61%)	118/138 86% (53%)	0.8745^†^

Number of T2 lesions, median (quartiles)	17 (7–38)	16 (6–36)	21 (8–41)	**0.0182**^‡^

At least one Gd-enhancing lesion – %	42%	42%	43%	0.9254^†^

At least one T1 hypointense lesion – %	68%	63%	73%	**0.0300**^†^

*Compares monofocal vs. multifocal patients. P-value in bold when p ≤ 0.05

^†^Fisher's exact test

^‡^Mann-Whitney U-test

EDSS: expanded disability status scale

CSF: cerebrospinal fluid

Gd: gadolinium

### Comparison of time to CDMS and MRI disease activity between the monofocal and multifocal patients (placebo patients)

Neither time to CDMS (Hazard ratio/± 95% CI: 1.09/0.70–1.71; p = 0.71) nor the annualized cumulative number of NALs (p = 0.47 by Wilcoxon test and p = 0.51 by baseline adjusted non-parametric ANCOVA, see Table 2) differed significantly between mono- and multifocal placebo patients.

**Table 2:** Disease course of monofocal vs. multifocal placebo patients after the CIS

	All	Monofocal	Multifocal	p-value*
N	176	93	83	

CDMS %* over 2 years	45%	47%	44%	0.7052^†^

Median annualized cumulative number of NALs over the study (quartiles)	3.2 (0.96–10.4)	3.0 (0.5–9.4)	3.6 (1.0–12.5)	0.4698^‡^

*Compares monofocal vs. multifocal patients

NAL: newly active lesion

CDMS: clinically definite MS

_† _Kaplan-Meier estimate at day 720, Log rank test

^‡^Wilcoxon test

### Comparison of the impact of MRI findings at screening, month 3, and month 6 on time to CDMS within the monofocal and multifocal group separately (placebo patients)

**Figure 1:**
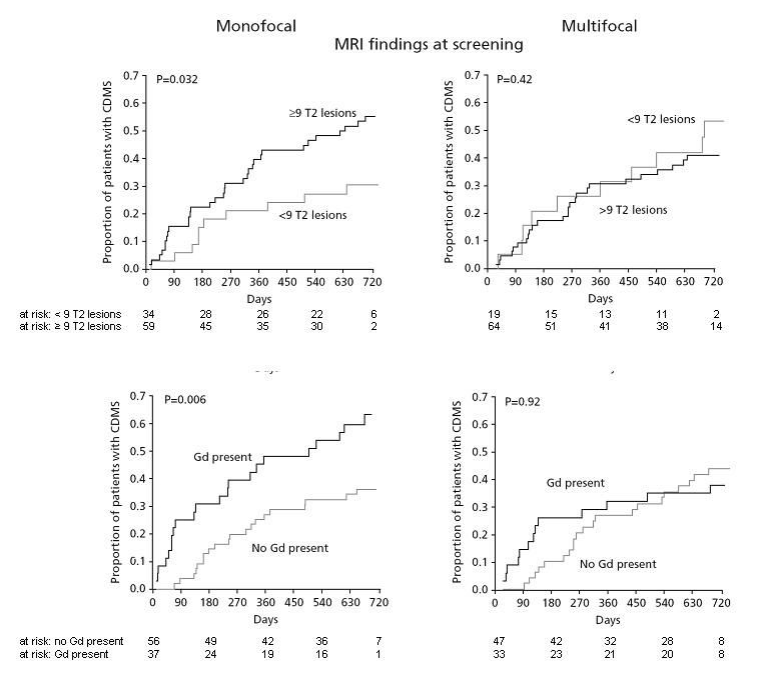
**Time to CDMS in mono- vs multifocal placebo patients stratified by MRI findings at screening**. Note the predictive value of baseline MRI findings in monofocal patients (left panels) and the absence of predictive value of MRI in multifocal patients (right panels). There was a significant interaction between mono-/multifocality and the presence of either ≥ 9 T2 hyperintense lesions (p = 0.042). CDMS: clinically definite MS.

**Figure 2:**
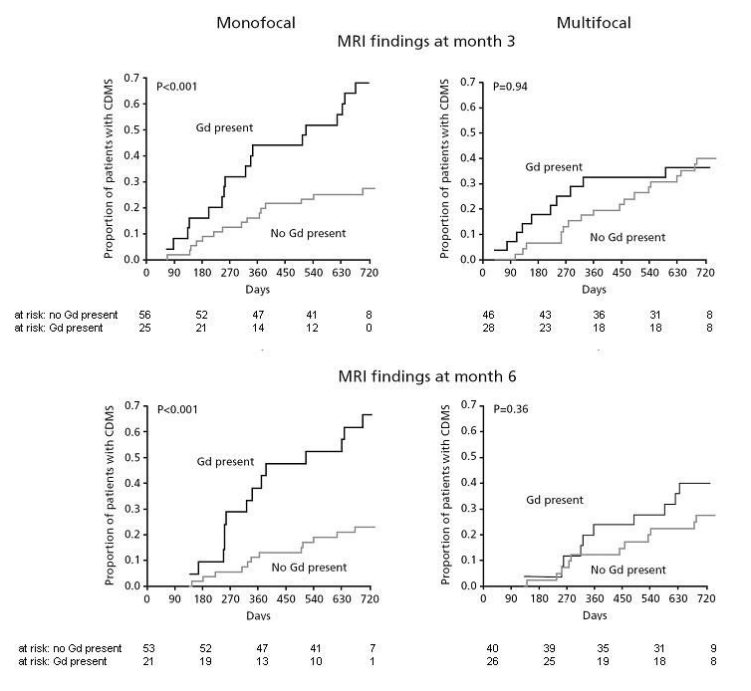
**Time to CDMS in mono- vs multifocal placebo patients stratified by MRI findings at month 3 and month 6**. Note the significant predictive value of months 3 and 6 MRI findings in monofocal patients (left panels) and the absence of predictive value in multifocal patients (right panels). There was a significant interaction between mono-/multifocality and the presence of at least one Gd-enhancing lesion at month 3 (both p = 0.042). CDMS: clinically definite MS.

The risk of CDMS was significantly higher in monofocal placebo patients with ≥ 9 T2-hyperintense lesions at screening (Hazard ratio/± 95% CI: 2.13/1.05–4.34; p = 0.032), with at least one Gd-enhancing lesion at screening (Hazard ratio/± 95% CI: 2.28/1.24–4.18; p = 0.006), with at least one Gd-enhancing lesion at month 3 (Hazard ratio/± 95% CI: 3.03/1.51–6.07; p < 0.002), and with at least one Gd-enhancing lesion at month 6 (Hazard ratio/± 95% CI: 3.98/1.84–8.65; p < 0.001) than in monofocal placebo patients without these criteria (Figure 1, 2).

The risk of CDMS was not significantly higher in multifocal placebo patients with ≥ 9 T2-hyperintense lesions at screening (Hazard ratio/± 95% CI: 0.74/0.36–1.54; p = 0.42), with at least one Gd-enhancing lesion at screening (Hazard ratio/± 95% CI: 0.96/0.48–1.93; p = 0.92), with at least one Gd-enhancing lesion at month 3 (Hazard ratio/± 95% CI:1.03/0.48–2.23; p = 0.94), and with at least one Gd-enhancing lesion at month 6 (Hazard ratio/± 95% CI: 2.04/0.86–4.86; p = 0.11), than in multifocal placebo patients without these criteria (Figure 1, 2).

This differential effect of MRI parameters on the risk of conversion to CDMS in these two patient groups (monofocal and multifocal) was confirmed by Cox proportional hazards regression. This analysis revealed a significant interaction between mono-/multifocality and either ≥ 9 T2-hyperintense lesions at screening (Hazard ratio/± 95% CI: 0.35/0.13–0.96; p = 0.042) or at least one Gd-enhancing lesion at month 3 (Hazard ratio/± 95% CI: 0.34/0.12–0.96; p = 0.042), but not between mono/multifocality and at least one Gd-enhancing lesion at screening (Hazard ratio/± 95% CI: 0.42/0.17–1.05; p = 0.064) or at least one Gd-enhancing lesion at month 6 (Hazard ratio/± 95% CI: 0.50/0.16–1.6; p = 0.246).

### Comparison of key baseline characteristics of multifocal patients by symptoms and multifocal patients by signs at the first event (all patients)

One hundred and twenty-two (55%) of the 222 multifocal patients presented by symptoms, while 100 (45%) presented by signs (the classification of these latter patients was based on the presence of signs indicating an additional clinical lesion, according to the central classification). Baseline characteristics did not differ significantly between multifocal patients by symptoms and by signs (Table 3).

**Table 3:** Disease characteristics of multifocal patients by symptoms and by signs at the CIS

	Multifocal	Multifocal by symptoms	Multifocal by signs	p-value
N	222	122	100	

Sex – % of females	76%	77%	74%	0.6389^†^

Age, median (quartiles)	31 (25–37)	30.5 (25–36)	31 (25–38)	0.7919^‡^

Steroid treatment – %	70%	72%	67%	0.4632^†^

EDSS (screening), median (quartiles)	2 1.5–2.5	2 1.5–2.5	2 1.5–2.5	0.9598^‡^

CSF positive of samples taken – %	118/138 86% (53%)	62/70 89% (51%)	56/68 82% (56%)	0.3405^†^

Number of T2 lesions, median (quartiles)	21 (8–41)	19.5 (8–47)	22.5 (9–40)	0.9130^‡^

At least one Gd-enhancing lesion – %	42%	45%	39%	0.3376^†^

At least one T1 hypointense lesion – %	73%	71%	74%	0.7627^†^

^†^Fisher's exact test

^‡^Mann-Whitney U-test

EDSS: expanded disability status scale

CSF: cerebrospinal fluid

Gd: gadolinium

### Comparison of time to CDMS and MRI disease activity between multifocal patients by symptoms and by signs (placebo patients)

There was no statistically significant difference between the survival curves of "Time to CDMS" comparing multifocal placebo patients by symptoms and by signs (Hazard ratio/± 95% CI: 0.72/0.36–1.45; p = 0.36). Multifocal placebo patients by symptoms developed a higher annualized number of NALs over the study period (p = 0.042 by Wilcoxon test; Table 4, supplemental data file).

**Table 4:** Disease course of multifocal placebo patients by symptoms and by signs after the CIS

	All multifocal	Multifocal by symptoms	Multifocal by signs	p-value*
N	83	50 (60%)	33 (40%)	
CDMS %* over 2 years	44%	49%	37%	0.3554^†^
				
Median annualized cumulative number of NALs over the study (quartiles)	3.6 (1.0–12.5)	5.3 (1.6–13.4)	2.6 (0–7.0)	0.0424^‡^

*Compares multifocal patients by symptoms vs. multifocal patients by signs

^†^Kaplan-Meier estimate at day 720, Log rank test

^‡^Wilcoxon test

CDMS: clinically definite MS

NAL: newly active lesion

Gd: gadolinium

## Discussion

In a cross-sectional analysis of baseline data from CIS patients in the BENEFIT study we have recently demonstrated that clinical dissemination of the disease corresponds to more widespread subclinical CNS pathology as detected by cerebral MRI[8]. In the present study we addressed whether clinical disease dissemination in these patients also indicates an increased risk for subsequent disease activity, and whether the presence versus absence of clinical dissemination has an impact on the prognostic value of MRI parameters.

Patients with monofocal versus multifocal clinical presentation did not differ in terms of their risk for CDMS or with respect to the annualized number of NALs over the 2-year placebo-controlled period, as has been reported previously [11]. However, we did find that MRI findings of subclinical disease dissemination or activity have a different prognostic value for development of CDMS in mono- versus multifocal CIS patients. The presence of at least nine T2 lesions or at least one Gd-enhancing lesion during screening was predictive for time to CDMS in monofocal patients though not in multifocal patients. Similar observations were made for the prognostic value of a new Gd-enhancing lesion on an MRI scan performed at month 3 or month 6. Thus, in monofocal, but not in multifocal patients the risk for CDMS depends on MRI findings. This differential impact of MRI findings in CIS patients with clinical monofocal versus clinical multifocal presentation was supported by a significant interaction between the impact of clinical mono-/multifocality and ≥ 9 T2 lesions at baseline and at least one Gd-enhancing lesion at month 3 on time to CDMS.

These findings strongly suggest that only in CIS patients with monofocal clinical presentation do MRI findings have prognostic value. We hypothesize that, whilst in monofocal CIS patients more pronounced subclinical disease dissemination might primarily reflect more active disease, similar findings in multifocal patients may be more indicative of prolonged subclinical disease evolution, and as such MRI adds less information in these patients.

To further elaborate on this hypothesis we expanded these comparisons to subgroups of multifocal patients: those by symptoms and those by signs, under the assumption that especially those patients multifocal by signs may have had an earlier event that was asymptomatic or forgotten, and therefore may have a longer and more benign form of the disease. We found similar baseline characteristics and only a nonsignificant difference in time to CDMS in these subgroups. The observation that multifocal placebo patients by symptoms tended to have more active MRI lesions during the study than multifocal placebo patients by signs further supports our hypothesis that the former may be considered more acute and at higher risk for future disease activity than a multifocal patient by signs in whom a longer subclinical disease history might be assumed. Differences with respect to patients showing at least one Gd-enhancing lesion (more in patients multifocal by symptoms) and patients showing at least one T1-hypointense lesion (more in patients multifocal by signs) as shown in Table 3, although not significant, are also supportive of our hypothesis.

We compared our results to those obtained in the Early Treatment of MS (ETOMS)[12] and Controlled High-Risk Avonex^® ^Multiple Sclerosis Prevention Study (CHAMPS)[13] studies, other interventional trials in CIS patients where comparable analyses were performed. In the ETOMS study, the presence of three or more MRI criteria as incorporated in the International Panel on the Diagnosis of Multiple Sclerosis guidelines[3] was also predictive for CDMS only in patients who were classified as clinically unifocal[6]. Unlike our observation, multifocal patients in ETOMS had a higher risk for CDMS. This difference in the predictive value of "multifocality" in the BENEFIT and the ETOMS cohort may result from the different methods used to classify patients in the two studies. Evaluation of clinical dissemination in ETOMS was based on the local investigator's assessment, whilst multifocality in BENEFIT was based on a central assessment procedure according to a proposed classification system of all presenting clinical symptoms and signs. Thus, multifocality in BENEFIT was also assumed in patients who, in addition to a monosymptomatic presentation [e.g. optic neuritis], presented with additional clinical signs (e.g. pyramidal dysfunction as indicated by extensor plantar response) indicating an additional clinical lesion. Also, in a post hoc analysis of the CHAMPS study[14] all patients were reclassified, taking into account the results of neurological examinations at baseline. In a multivariate analysis, classification by focality was not predictive of conversion to CDMS, which is in line with our results.

All patients in BENEFIT, ETOMS, and CHAMPS had a minimum number of asymptomatic T2 lesions; therefore it is unclear whether these results also can be applied to CIS patients with fewer or no lesions. Further limitations of our analyses should be considered. All subgroup analyses were performed post hoc and our results need confirmation, particularly the novel findings in the subgroups of multifocal CIS patients. However, we would like to emphasize the similarities between our findings and the ETOMS study[6] in terms of the lack of impact of MRI findings in multifocal patients on the risk of CDMS.

## Conclusion

To summarize, MRI lesions may generally be interpreted as indicators of past and future disease activity in patients with monofocal presentation, though not in multifocal patients, in whom their presence does not add to the risk as defined by the clinical evaluation only. Our findings show that a carefully performed neurological assessment of symptoms and signs in CIS patients is important to define the risk of conversion to CDMS and the potential added value of MRI investigations.

## Competing interests

This study was sponsored by Bayer Schering Pharma AG, Berlin, Germany. Dr Nielsen has nothing to disclose. Dr Pohl has received personal compensation for activities with Schering AG as an employee. Consultancy for Schering, Aventis, UCB, Roche, Serono, Novartis. Dr Freedman has received personal compensation for activities with Bayer-Schering Pharmaceuticals, Merck-Serono, Pfizer Inc, Teva Neuroscience, Biogen Idec, Genentech, Inc. and BioMS as a consultant and advisory board member. Dr Edan has nothing to disclose. Prof Miller has received personal compensation for activities with Biogen Idec, GlaxoSmithKline, Inc., and Schering AG as a consultant. Prof Miller has received personal compensation in an editorial capacity for Journal of Neurology. Prof Miller has received research support from Biogen Idec., GlaxoSmithKline, Inc., and Schering AG. Dr Kappos has nothing to disclose. Dr Bauer has received personal compensation for activities with Schering AG as an employee. Dr Rupert Sandbrink received personal compensation from Schering AG as a salaried employee of this company. Prof Polman has received consulting fees from Biogen Idec, Schering AG, Teva, Serono, Novartis, GlaxoSmithKline, Novartis, and Teva. In the past year, dr. Uitdehaag has received compensation for consultancy from Novartis and Merck Serono. In the past year, dr. Uitdehaag received personal compensation from Ariez Medical Publishing for serving as a jounal editor. The institute for which dr Uitdehaag works received financieal support for research activities from Biogen Idec, Bayer Schering Pharma, GlaxoSmithKline, Novartis, Merck Serono and Teva.

## Authors' contributions

JN drafted the manuscript, performed the statistical analysis and participated in the design of the study, CP participated in the design and coordination of the study, drafted the manuscript and performed the statistical analysis, CHP participated in the design and coordination of the study, and drafted the manuscript, FB commented on the manuscript and participated in the design and coordination of the study, MF commented on the manuscript and participated in the design and coordination of the study, GE commented on the manuscript and participated in the design and coordination of the study, DM commented on the manuscript and participated in the design and coordination of the study, LB commented on the manuscript and participated in the design and coordination of the study, RS commented on the manuscript and participated in the design and coordination of the study, LK commented on the manuscript and participated in the design and coordination of the study, BU drafted the manuscript, performed the statistical analysis and participated in the design of the study. All authors read and approved the final manuscript.

## Pre-publication history

The pre-publication history for this paper can be accessed here:


