# Correction: Prognostic implications of a carefully performed neurological assessment in patients with a first event suggestive of multiple sclerosis

**DOI:** 10.1186/1471-2377-11-19

**Published:** 2011-02-04

**Authors:** Jessica M Nielsen, Christoph Pohl, Chris H Polman, Frederik Barkhof, Mark S Freedman, Gilles Edan, David H Miller, Lars Bauer, Rupert Sandbrink, Ludwig Kappos, Bernard MJ Uitdehaag

**Affiliations:** 1 MS Center, Department of Neurology, VU Medical Center, Amsterdam, The Netherlands; 2 Bayer Schering AG, Berlin, Germany; 3 Department of Radiology, VU medical Center, Amsterdam, The Netherlands; 4 Department of Neurology, The Ottawa Hospital, Ontario, Canada; 5 Department of Neurology, Hopital Pontchaillou, Rennes, France; 6 Department of Radiology, Queen square hospital, London, UK; 7 Department of Neurology, Kantonsspital, Basel, Switzerland; 8 Department of Epidemiology and Biostatistics, VU Medical Center, Amsterdam, The Netherlands

## 

In our article [1], we did not declare some potential competing interests for Prof. Dr. med. Ludwig Kappos:

Declaration of competing interests:

Prof. Dr. med. Ludwig Kappos

LK has participated in the last 24 months as principal investigator, member or chair of planning and steering committees or advisory boards in corporate-sponsored clinical trials in multiple sclerosis and other neurological diseases. The sponsoring pharmaceutical companies for these trials include Actelion, Advancell, Allozyne, BaroFold, Bayer Health Care Pharmaceuticals, Bayer Schering Pharma, Bayhill, Biogen Idec, BioMarin, CLC Behring, Elan, Genmab, GeNeuro SA, Genmark, GlaxoSmithKline, Lilly, Merck Serono, MediciNova, Novartis, Novonordisk, Peptimmune, sanofi-aventis, Santhera, Roche, Teva, UCB and Wyeth.

I have also lectured at medical conferences or in public on various aspects of the diagnosis and management of multiple sclerosis. In many cases these talks have been sponsored by non-restricted educational grants from one or another of the above listed companies.

Honoraria and other payments for all these activities have been exclusively used for funding of research of my department.

Research and the clinical operations (nursing and patient care services) of the MS Center in Basel have been supported by non-restricted grants from one or more of these companies and by grants from the Swiss MS Society, the Swiss National Research Foundation, the European Union, the Gianni Rubatto, Novartis and Roche Research Foundations.

## Pre-publication history

The pre-publication history for this paper can be accessed here:

http://www.biomedcentral.com/1471-2377/11/19/prepub
